# Leveraging MDS2 and SBOM data for LLM-assisted vulnerability analysis of medical devices

**DOI:** 10.1016/j.csbj.2025.07.012

**Published:** 2025-07-17

**Authors:** Stefan Stein, Michael Pilgermann, Simon Weber, Martin Sedlmayr

**Affiliations:** a Department of Computer Science and Media, Brandenburg University of Applied Sciences, Magdeburger Straße 50, Brandenburg an der Havel, 14770, Brandenburg, Germany; b Department of Computer Science, Heinrich-Heine-University Dusseldorf, Universitätsstraße 1, Düsseldorf, 40225, North Rhine-Westphalia, Germany; c Institute for Medical Informatics and Biometry, Dresden University of Technology, Fetscherstraße 74, Dresden, 01307, Saxony, Germany

**Keywords:** Cyber security, IT security, Large Language Model (LLM), Manufacturer disclosure statement for medical device security, MDS2, Medical cyber-physical systems, Medical device, Medical information system, Operational technology (OT) security, Vulnerabilities

## Abstract

This study investigated the use of a semi-automated, Retrieval-Augmented Generation (RAG)-based multi-agent architecture to analyze security-relevant data and assemble specialized exploitation paths targeting medical devices. The input dataset comprised device-specific sources, namely, the Manufacturer Disclosure Statement for Medical Device Security (MDS2) documents and Software Bills of Materials (SBOMs), enriched with public vulnerability databases, including Common Vulnerabilities and Exposures (CVE), Known Exploited Vulnerabilities (KEV), and Metasploit exploit records. The objective was to assess whether a modular, Large Language Model (LLM)-driven agent system could autonomously correlate device metadata with known vulnerabilities and existing exploit information to support structured threat modeling. The architecture follows a static RAG design based on predefined prompts and fixed retrieval logic, without autonomous agent planning or dynamic query adaptation. The developed Vulnerability Intelligence for Threat Analysis in Medical Security (VITAMedSec) system operates under human-prompted supervision and successfully synthesizes actionable insights and exploitation paths without requiring manual step-by-step input during execution. Although technically coherent results were obtained under controlled conditions, real-world validation remains a critical avenue for future research. This study further discusses the dual-use implications of such an agent-based framework, its relevance to patient safety in medical device cybersecurity, and the broader applicability of the proposed architecture to other critical infrastructure sectors. These findings emphasize both the technical potential and ethical responsibility for applying semi-automated AI workflows in medical cybersecurity contexts.

## Graphical abstract

**Figure:**
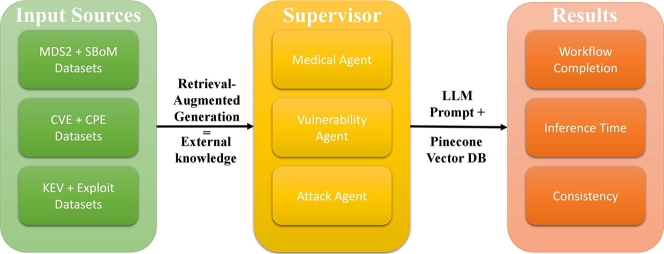

## 1 Introduction

Since the introduction of commercially viable Large Language Models (LLMs) in 2020, these systems have rapidly evolved and are now playing an increasingly central role in cybersecurity. Early transformer-based architectures such as Google's BERT laid the foundation for scalable language understanding [1]. The release of GPT-3 by OpenAI has marked a significant shift in its capability and accessibility, transforming LLMs from experimental tools to general-purpose technologies applicable in areas ranging from automated text generation to complex security analysis [2]. The subsequent launch of ChatGPT by the end of 2022 accelerated the adoption of LLMs across research, industry, and education, according to Zong et al. [3]. Major competitors such as Google's Bard (now Gemini) and Anthropic's Claude, alongside powerful open-source models like Meta's Llama and Mistral, have fostered a dynamic ecosystem that encourages exploration of new application domains. In cybersecurity, LLMs are increasingly applied to tasks such as real-time threat detection, automatic vulnerability analysis, and code-based exploit identification [4], [5], [6], [7]. Examples include VulnHuntR, which demonstrates autonomous multilayered vulnerability identification through code flow analysis [8], [9], and AIBugHunter, which shows vulnerability classification and automated remediation suggestions [10]. These efforts have contributed to the broader trend of integrating AI into security operations to create adaptive, scalable, and context-aware defense systems. Parallel developments focused on LLM-driven autonomous agents for cybersecurity [11], [12], [13]. Such agents can independently scan systems, coordinate responses to distributed attacks, and propose mitigation measures [14]. However, these technologies also raise concerns regarding their dual-use nature, and studies like EvilModel 2.0 have demonstrated how AI models can embed undetectable malware [15], and LLM-driven phishing campaigns have outperformed human-crafted efforts in linguistic quality and deception success [16]. Fang et al. demonstrated in 2024 that GPT-4 can autonomously exploit known vulnerabilities with high success rates, surpassing traditional vulnerability scanners [17], [18]. These findings highlight both the promise and risk of LLM-based cybersecurity tools, particularly in high-stakes domains such as healthcare. Medical devices are increasingly network-connected, standardized, and thus, vulnerable to cyberattacks [19]. A successful compromise could endanger patient safety, for example, by manipulating infusion pumps [20], [21] or corrupting imaging systems [22], [23]. Although security-relevant information is formally documented in Manufacturer Disclosure Statements for Medical Device Security (MDS2), such information is often underutilized in active threat modeling. To address this gap, this study investigated the use of a semi-automated RAG-enhanced multi-agent architecture for assembling structured exploitation paths targeting medical devices while operating under human supervision. The developed Vulnerability Intelligence for Threat Analysis in Medical Security (VITAMedSec) system aims to contribute to the emerging field of AI-assisted medical cybersecurity by safely exploring vulnerability linkages and attack path generation. It is important to note that, in this study, no model training was performed; instead, RAG was used to dynamically augment the model's input context with external knowledge.

## 2 Related work

As first analyzed by Stein et al. in 2024, MDS2 documents contain detailed information about a device's communication protocols, encryption settings, authentication mechanisms, and patch management strategies [24]. When MDS2 data are combined with structured vulnerability sources, such as CVE [25], Common Platform Enumeration (CPE) [26], KEV [27], and Software Bills of Materials [28], LLMs can construct detailed threat models that enable the simulation of exploitation paths and the identification of system weaknesses for specific medical devices. Shen (2025) introduced a related line of research that leveraged MDS2 security attributes and the MITRE ATT&CK framework to enhance cyber risk assessment for network-connected medical devices [29]. His approach mapped device-specific vulnerabilities extracted from MDS2 documents onto relevant ATT&CK tactics and techniques, enabling structured, healthcare-focused threat modeling. However, the methodology remains centered on static risk evaluation without exploring dynamic exploitation path construction or agent-based simulation workflows. Building on this foundational work, this study advances the field by operationalizing structured vulnerability information for the semi-automated assembly of targeted exploitation scenarios within a multi-agent RAG-enhanced framework. Initial implementations, such as Adithya's vulnerability scanner [30], focused on CPE-CVE matching to enhance asset-based risk assessments. Ghosh et al. recently introduced CVE-LLM in 2024, using LLMs to link asset metadata to known attack patterns and vulnerabilities in medical systems [31]. Xu et al. (2024) and Han et al. (2024) provide an overview of LLM use in cybersecurity, highlighting semantic threat detection and vulnerability reasoning across unstructured datasets [32], [33]. Recent studies, such as Motlagh et al. (2024) [34] and Kouremetis et al. (2025) [35], have shown that LLMs perform well in defensive tasks, such as threat detection or incident response, while their offensive capabilities (e.g., attack planning and payload synthesis) are still evolving. For example, the Operational Evaluation Framework for Cyber Security Risks in AI (OCCULT) framework benchmarks models such as DeepSeek-R1, Llama, and Mixtral in realistic offensive scenarios, and reveals differences in performance across planning, code generation, and automated exploitation tasks [35]. Recent policy frameworks highlight the growing dual-use risks of AI models in critical sectors such as healthcare. The NIST AI 800-1 guideline emphasizes that dual-use AI systems can enhance cybersecurity and facilitate cyberattacks on medical infrastructure, such as infusion pumps and imaging systems, necessitating strict risk-mitigation measures including access restrictions and data filtering [36]. Similarly, the Johns Hopkins Center for Health Security underscores the potential of AI to accelerate cyberattacks on hospital IT systems while simultaneously enabling improved defensive capabilities through predictive analytics, calling for robust governance frameworks for AI in healthcare environments [37]. Although previous studies have demonstrated the use of LLMs for static vulnerability evaluation and semantic threat detection, they have largely relied on pre-ingested knowledge. Recent developments in Retrieval-Augmented Generation aim to overcome this limitation by enabling runtime access to external databases for contextually enriched responses in security-related applications although they have primarily focused on defensive tasks [38], [39]. Despite its growing relevance, the systematic use of RAG-driven architectures for the semi-automated assembly of exploitation paths, particularly in regulated medical environments, remains largely unexplored. This study extends existing research by designing, implementing, and evaluating a semi-automated, multi-agent, RAG-based workflow that correlates device metadata with vulnerability and exploit information to generate plausible exploitation scenarios under human supervision. The proposed framework connects structured device documentation with public vulnerability intelligence through LLM-assisted reasoning, thereby enabling the safe investigation of cybersecurity risks in medical environments. Moreover, the modular design of the architecture facilitates adaptation across other critical infrastructure domains, while maintaining a focus on responsible and ethical deployment.

## 3 Methodology

In this study, four research questions were defined to explore how LLMs can be used to analyze security-relevant data sources, including CVE, CPE, KEV, SBOM, attack payloads and MDS2 documents, within the domain of medical device cybersecurity. The methodology is designed to implement an agent-based approach, in which specialized trained AI agents autonomously handle different security tasks. This structured approach enables distributed and adaptive analysis, optimizing the assessment of offensive and defensive cybersecurity challenges in the medical environment.

### 3.1 Research questions

#### 3.1.1 RQ 1 - are MDS2 and SBOM data complete and sufficiently structured to be used as input for LLM-based vulnerability analysis?

This research question investigates whether and how security-relevant data from MDS2 documents (especially Version 2019), manufacturer documentation and security whitepaper can be structured for an agent-based processing approach to medical device cybersecurity. Previous research has confirmed that these documents contain essential security information, including vulnerabilities, authentication mechanisms, and network configurations, but require significant preprocessing for automated analysis (Stein et al., 2024). To achieve this, the study employed a structured parsing and normalization pipeline implemented in Python, utilizing libraries such as PyMuPDF for PDF text extraction and csv and re for downstream processing. MDS2 documents (version 2019) were processed in their original PDF format using a layout-aware text parser that preserved the line integrity and section order. A reference file listing all 216 official MDS2 field identifiers and their associated question texts was prepared in pipe-delimited CSV format. During parsing, each field identifier (e.g., PAUT-1) is used to locate its corresponding segment within the text with the search bounded by the next known field code. This enables robust segmentation of content, even in noisy or scanned document variants. Regular expressions were applied to identify categorical responses such as Yes, No, N/A, and See Note, with precedence given to the first occurrence following a question mark to avoid misclassification from the surrounding context. The extracted answers were written to a row-based tabular output, preserving column alignment with the reference schema. Each output file contained one row per device and one column per MDS2 attribute, allowing downstream tools to sort, compare, and filter responses across devices. Where multiple valid values were detected (e.g., answer and additional values), a secondary flag (Multivalue) was added to an adjacent cell. An example summary row for CSV exports is as follows:

**Figure:**
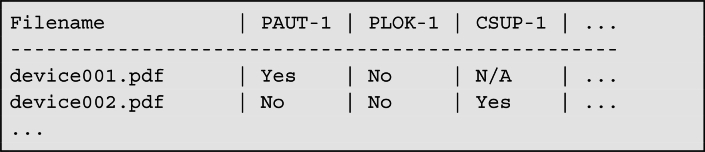

The resulting corpus of normalized, tabular MDS2 data formed the input to a retrieval-augmented system that enabled LLM agents to query structured device properties consistently. This CSV-based representation also allowed easy enrichment with SBOM attributes, CPE identifiers, and threat intelligence from the CVE and KEV datasets without relying on format transformations or external database layers. First, structured data were utilized within a retrieval-augmented framework to provide uncensored LLMs with enriched contexts, enabling them to operate as part of an agent-based system in conjunction with other supervised agents (3.1.3 and 3.1.4).

The dataset analyzed in this study consisted exclusively of authentic MDS2 documents and SBOMs obtained from real-world medical devices through manufacturer cooperation agreements and authorized sources. These documents were complemented by manufacturer-specific whitepapers that provided valuable additional insights, including descriptions of the intended operational environment, implemented security controls, lists of open ports, and details of running services. No synthetic or artificially generated data were used to fill these gaps; all analyses were based solely on genuine documentation to ensure realistic and representative results.

#### 3.1.2 RQ 2 - can public vulnerability and exploit sources be integrated to enrich device-specific data for LLM-based threat modeling?

To address RQ2, a structured and data-driven approach was developed to construct a semantically enriched dataset for LLM-based analysis within the domain of medical device cybersecurity. The objective was to enrich an existing database with information on vulnerabilities, active exploitation, and payload techniques. Four primary data categories were integrated: CVE, KEV, payloads, and unstructured security sources. The CVE data provided standardized information on publicly disclosed vulnerabilities, including identifiers, severity scores, descriptions, publication dates, affected products, and reference links. CPE-formatted product identifiers enable the precise mapping of relevant platforms. These structured data form the foundation for semantic enrichment and correlation. KEV data introduced operational threat intelligence by associating known vulnerabilities with real-world exploitation. Records included metadata such as exploitation dates, attack techniques based on the MITRE ATT&CK framework, and remediation guidance. This layer supports prioritization and realistic threat modeling for security-related applications. Payload data collected from public exploit repositories were used to bridge the gap between vulnerability and executable attack logic. Payloads were categorized by technique, architecture, and target platform, and included metadata such as scripting format, required parameters, and optional links to associated vulnerabilities. This dataset enhances the ability of the system to reason for practical exploitation scenarios. Unstructured sources such as technical documentation, hardening guides, and network schematics were analyzed to extract contextual information on device architectures, security mechanisms, and potential entry points. Relevant content was converted into a structured form for integration with the core dataset. All data were normalized, transformed, and semantically processed to ensure compatibility with the LLMs. The enriched machine-readable dataset allows for the automated association of vulnerabilities with specific system components, supporting the development of a specialized LLM capable of threat modeling, vulnerability analysis, and attack pattern extraction in the context of medical device cybersecurity.

#### 3.1.3 RQ 3 - are LLMs capable of generating plausible attack scenarios for medical devices based on enriched vulnerability data?

**LLM Evaluation**

Before evaluating whether a specially trained LLM can generate and assemble exploitation paths for medical devices, a model selection phase was first conducted. This step aims to identify the LLMs with the highest potential for offensive cybersecurity tasks, particularly in automated vulnerability exploitation and attack vector generation. To ensure a solid foundation for subsequent experiments, this study compared the performance of several available LLMs under controlled conditions. The evaluation included non-fine-tuned open-source models (e.g., Llama and DeepSeek) and fine-tuned security-focused variants, such as Qwen2.5-14B-CyberSecurity, Dolphin3.0-Llama3.2-3B-Q4_K_M-GGUF, and Llama-3-WhiteRabbitNeo-8B-v2.0. Only models capable of local execution, without reliance on external APIs, were considered. In addition, the selection was limited to models with fewer than 70 billion parameters, to ensure compatibility with consumer-grade hardware. Preference was given to models that were either fine-tuned on security-relevant datasets or demonstrated strong performance on cybersecurity benchmarks. Each model was tested using two domain-specific queries to evaluate its reasoning over structured medical device data and semantically enriched vulnerability information, directly addressing Research Questions 1 and 2. The first query, *“Given a device SBOM containing OpenSSL 1.0.1, suggests possible exploit vectors.”* tested the ability of the model to interpret a simplified SBOM reference as a contextual input, rather than processing a full real SBOM, and to link identified vulnerabilities (RQ1) with external knowledge sources such as CVEs and KEVs (RQ2). The second query, *What steps would follow after initial RCE on a PACS server to exfiltrate DICOM files?”* evaluated the model's ability to synthesize realistic multistage attack paths based on known exploitation tactics and domain-specific contexts. Responses were assessed for technical correctness, logical coherence, and contextual appropriateness, including relevant details such as protocol use, file formats, and access methods. Additional evaluation criteria included reasoning accuracy, prompt adherence, and the model's ability to use embedded threat intelligence. All the tests were conducted in isolated, non-networked environments to ensure safe and ethical experimentation.

**Retrieval-Augmented Generation as external knowledge base**

Building on the LLM selection, the next phase of the study explored whether these models could operate effectively in a retrieval-augmented setting to semi-automated assembling exploitation paths or identify novel attack vectors in medical environments. To ensure a transparent and controlled evaluation environment, a methodology based on static prompt injection, combined with structured retrieval, was employed. This approach allows the selected LLMs to operate on enriched, context-aware data, while preventing uncontrolled generation or multistep reasoning beyond the scope provided. This approach is grounded in the principle of RAG, and was selected to enable a controlled and transparent evaluation of the LLM's capabilities when exposed to domain-specific, security-relevant contextual data. The methodological foundation was formed by a curated dataset developed as a part of prior research that integrated information from manufacturer documentation, MDS2 files, public vulnerability databases, and exploit repositories. This dataset includes metadata on medical devices, component-level software inventories in the form of Software Bills of Materials, Common Platform Enumeration tags derived from SBoMs, vulnerability records, real-world attack evidence drawn from KEV catalogs, and open-source payload collections. The system architecture is designed to retrieve context passages from a vector database containing integrated data. These passages were selected based on their relevance to a given test scenario, such as generating an exploit code for a specific vulnerable device, and were dynamically inserted into the prompt along with task formulation. The language model, running without additional fine-tuning, was then queried to synthesize an output using only this contextual knowledge and its pretrained reasoning abilities. Chunking strategies and metadata filters were employed to ensure that the context presented to the model was concise, technically relevant, and semantically consistent. This method was chosen over more complex alternatives such as recursive retrieval or agent-based orchestration for several reasons. Most importantly, they offer a high degree of control and reproducibility. Each input to the model is fully defined and each output can be directly traced back to its supporting context. This is particularly crucial when evaluating a model's potential for harmful or dual-use generation in which transparency and interpretability are essential for ethical oversight. In contrast to autonomous agents, which introduce multistep decision logic and dynamic context construction, the static RAG approach maintains a single-pass architecture, which limits the risk of uncontrolled content generation. Furthermore, this method aligns with the constraints of the unfine-tuned LLM used in the study. By avoiding additional training, the observed model behavior can be attributed to the interaction between the pretrained weights and the external knowledge supplied at runtime. This isolation of variables simplifies the analysis and clarifies whether the generative capabilities of the model are inherently dangerous when combined with the openly available domain-specific contexts. Overall, the static prompt injection method with structured retrieval represents a low-complexity and high-fidelity strategy for exploring the security implications of LLMs in the context of medical devices. It enables rigorous, ethically manageable experimentation, and provides a defensible baseline for assessing whether such systems can indeed produce functional, targeted malware in an appropriate context.

#### 3.1.4 RQ 4 - how effectively can a semi-automated RAG workflow with AI agents generate targeted attack paths for medical devices?

To answer the research question of whether an automated workflow for the autonomous generation of specialized malicious code targeting network-enabled medical devices can be realized using Retrieval-Augmented Generation and AI agents, this study follows a structured, multistage approach. The objective was to design and evaluate a modular system in which dedicated AI agents collaborate to identify vulnerabilities, correlate them with attack vectors, and generate device-specific payloads with minimal human intervention. In the initial phase, relevant data were collected and processed from multiple sources, including Software Bills of Materials, MDS2 documents, CVE and CPE databases, KEV records, and public exploit repositories such as Metasploit [40]. Heterogeneous data were preprocessed using optimized parsers, converted into a standardized format, segmented into context-preserving chunks (e.g., 500–1000 tokens), and stored in topic-specific vector databases. Before deployment, each vector database was semantically enriched using metadata annotations and periodically updated using automated pipelines with live data from public sources, to ensure that the retrieval operations remained current and accurate. This setup enabled real-time access to high-quality context data without requiring fine-tuning of the underlying language models. The system architecture foresees a division of responsibilities across three specialized AI agents, each of which operates on its own domain-specific vector store using RAG-based retrieval.

• Agent 1 handles information extraction and retrieval from MDS2 documents and SBoMs, focusing on device structure, software stack, and network exposure.
• Agent 2 processes vulnerability-related data, including CPE identifiers, CVEs, and associated severity metrics.
• Agent 3 focuses on KEV entries and payload metadata, drawing links between real-world exploits, known tactics, and potential code reuse.

These three agents are coordinated by a supervisory agent that orchestrates multistep interactions using techniques such as query planning, metadata-based routing, and recursive cross-agent retrieval. The supervisor integrates intermediate results, resolves ambiguity across data sources, and issues function calls to external APIs and web scraping modules when required; for example, to fetch real-time device configurations or exploit examples from open web sources. Throughout the workflow, agents apply Retrieval-Augmented Generation to construct queries dynamically, access their respective vector databases, and collaboratively build coherent outputs. These may include threat narratives, attack chains, or functional exploitation templates tailored to the device under investigation. In the final evaluation step, the feasibility of the architecture is assessed in terms of automation, performance, and risk. This included analyzing the extent to which the end-to-end process could operate without human guidance, and examining the ethical and technical implications of autonomous exploit generation. The result of this methodology is a fully documented system architecture featuring a distributed agent model with real-time RAG integration, support for external data enrichment via API and web scraping, and modular extensibility for further task specialization.

### 3.2 Research procedure

**Fig. 1:**
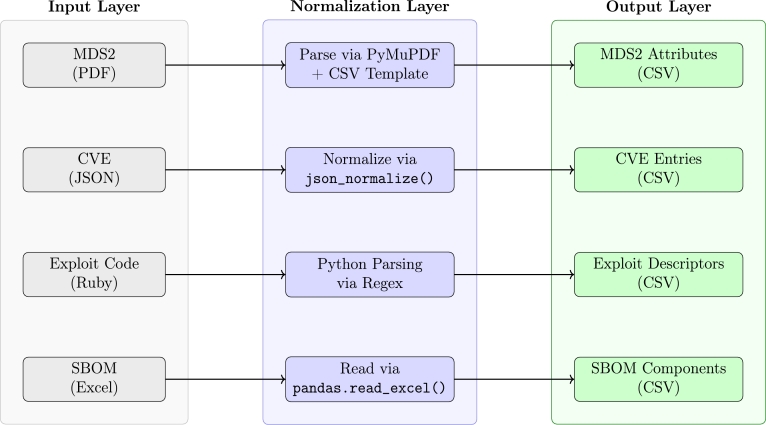
Overview of preprocessing workflows to extract structured CSVs from unstructured or semi-structured security sources.

To operationalize the four research questions (RQ1–RQ4), the study followed a stepwise research procedure divided into four consecutive phases. Each phase addressed a specific question and corresponded to a distinct methodological step as outlined below. The first phase of the research procedure, addressing RQ1, focuses on building a reproducible pipeline to extract structured attributes from heterogeneous security documentation. These included MDS2 forms in PDF format, SBOM spreadsheets (Excel), CVE threat entries (JSON), and exploit definitions written in Ruby. Each document type was processed using dedicated Python-based scripts, incorporating libraries such as PyMuPDF, pandas, csv, and re for format-specific parsing and normalization. As illustrated in Fig. 1, the extraction process was organized into three conceptual layers: (i) an input layer handling ingestion of raw files, (ii) a normalization layer applying syntax-aware transformations, and (iii) an output layer that produces aligned tabular CSV files ready for downstream retrieval workflows. The implementation details of the parsing scripts, agent configurations, and example outputs that underpin this workflow and ensure reproducibility are provided in a public GitHub repository [41]. For example, a scanned MDS2 form from an ultrasound system was parsed into 216 structured attributes using the extraction pipeline. Notably, the attribute PAUT-1 (Person Authentication) was successfully extracted and assigned the value Yes using pattern-based parsing techniques. While the majority of fields were processed automatically, technical details such as port numbers and communication protocols were manually identified and compiled by the authors owing to inconsistent formatting. The resulting structured corpus served as retrieval-optimized input for downstream processing, forming the backbone of the RAG-based LLM system without requiring model fine-tuning. Building on the normalized device data, the next step addressed RQ2 by semantically enriching the dataset using external threat intelligence. This involved linking the SBOM-derived CPE identifiers to publicly available CVE and KEV records. To complement this threat mapping, exploit modules from the Metasploit framework were parsed to extract CVE references and identify the relevant target platforms. Together, the outputs of Phases 1 and 2 resulted in a semantically enriched, structured knowledge base that provided high-context input to the downstream LLMs without requiring model weight modification. To assess model performance in Phase 3 (RQ3), a standardized evaluation protocol was implemented. Each LLM was presented with identical prompt contexts derived from the structured knowledge base and was assessed across multiple trials. The output quality was measured using a three-dimensional rubric: *technical accuracy*, *logical coherence*, and *level of detail*. Two independent reviewers annotated the responses in a blinded manner, enabling consistent scoring across models. The resulting evaluation matrix forms the basis for cross-model comparisons and guides the selection of agents in the final workflow. The decision to adopt a flat CSV-based data model for all intermediate representations rather than using a semantic graph or database backend was motivated by transparency, reproducibility, and minimal infrastructure dependencies. This design choice ensures that each transformation step can be executed and validated independently without requiring additional storage layers, which is particularly relevant for security-focused workflows that prioritize auditability and isolation. The final phase (RQ4) explored automation potential through agentic implementation of the RAG paradigm. To this end, a set of specialized agents was developed, each responsible for a discrete subtask, such as device information extraction (W_Medical), vulnerability mapping (W_Vuln), and payload suggestions (W_Attack). These agents operated over previously structured CSV data and utilized contextual retrieval to answer task-specific prompts. Although orchestration was primarily manual for security reasons, each agent's performance was evaluated by verifying its output against known device configurations and CVE references. The modular design allows for chained execution, enabling multistage threat modeling workflows under controlled execution. All models were evaluated in isolated sandbox environments on commercially available hardware, ensuring realistic deployment without the need for specialized AI infrastructure. The experiments followed dual-use research guidelines, including internal reviews and controlled handling of the generated outputs. The results of this evaluation informed the model selection for subsequent experiments involving contextual retrieval and adversarial generation in the domain of medical-device cybersecurity.

## 4 Results

For the research, the content of 210 MDS2 questionnaires from the 2019 version was combined with information from 161 security white papers, and this combination was used as a preliminary basis. During the course of the investigation, the MDS2 questionnaires were supplemented by SBoM entries combined with the records of CVEs, CPEs, KEVs, and the content of Metasploit modules prepared on test day March 16, 2025.

### 4.1 RQ 1 - are MDS2 and SBOM data complete and sufficiently structured to be used as input for LLM-based vulnerability analysis?

#### 4.1.1 MDS2

The extraction of questions and answers was carried out using pre-existing algorithms and scripts originating from earlier research efforts, which were systematically adapted for the purposes of this study. As noted in previous research, some documents contained formatting errors owing to the lack of implementation of standardization requirements. As part of evaluating the suitability of MDS2 documents as a foundational source for training Large Language Models on security-relevant medical device data (RQ1), 210 documents were systematically assessed. The analysis focused on four key categories defined in the MDS2 specification: Software Bill of Materials (SBOM), System Hardening (SAHD), Security Guidance (SGUD), and Person Authentication (PAUT), each represented by specific standardized questions. As shown in Table 1, the SBOM-related items — “Does the system maintain a list of software components?” (SBOM-1), “Does the vendor make a list of software available to customers?” (SBOM-2), and “Are software versions maintained for each component?” (SBOM-2.1), demonstrating high coverage. SBOM-1 and SBOM-2.1 received affirmative responses in 199 and 197 documents, respectively. These structured software inventories can be converted into formats such as Common Platform Enumeration, enabling linkage to public vulnerability datasets such as CVE and KEV. Is security guidance adequately reflected in manufacturer-provided documentation (SGUD-1), and are vendor recommendations for the system configuration actually implemented (SGUD-4)? SGUD-1 was answered “Yes” in 196 documents and SGUD-4 in 118 documents. These entries provide a valuable context for threat modeling and defense evaluations. In contrast, “Can the device be hardened beyond the default provided state?” (SAHD-14) and “Are instructions available from vendor for increased hardening?” (SAHD-14.1) showed greater variability with 79 and 57 positive responses, respectively. However, even partial coverage allows LLMs to incorporate system-level security postures into simulations or analytical tasks. The question is whether the system implements user-authentication mechanisms. PAUT-4 received 144 “Yes” responses, indicating consistent documentation of access control, which is a critical factor for modeling privilege escalation or lateral movement. Overall, the MDS2 documents exhibit structured, machine-readable content across all key security domains. When properly parsed and normalized, they form a robust foundation for training or prompting LLMs to generate realistic device-specific attack scenarios. These findings support the hypotheses formulated in RQ1.

**Table 1:** MDS2 Sections Regarding Security-Relevant Information (N = 210).

Category	Item	Yes	No	Other^a^	Total
SBOM	SBOM-1	199	8	2	210
	SBOM-2	97	100	13	210
	SBOM-2.1	197	1	12	210

Hardening	SAHD-14	79	101	30	210
	SAHD-14.1	57	102	51	210

Security Guidance	SGUD-1	196	12	2	210
	SGUD-4	118	76	16	210

Person Authentication	PAUT-4	144	52	14	210

a The “Other” column represents the sum of the original *Not found*, *N/A*, and *See Note* responses.

#### 4.1.2 SBoMs

**Fig. 2:**
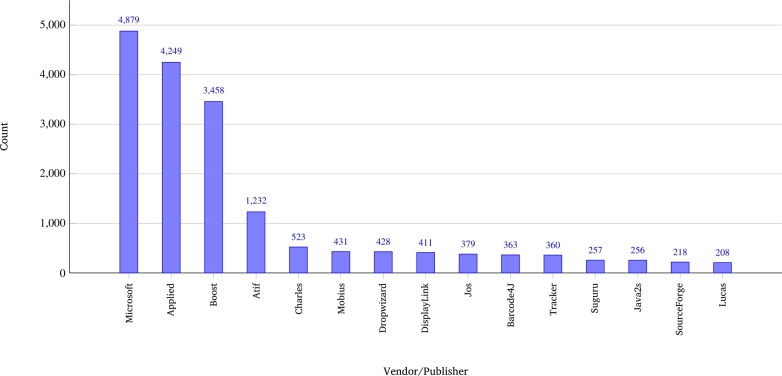
Top 15 software vendor/publisher mentions.

**Fig. 3:**
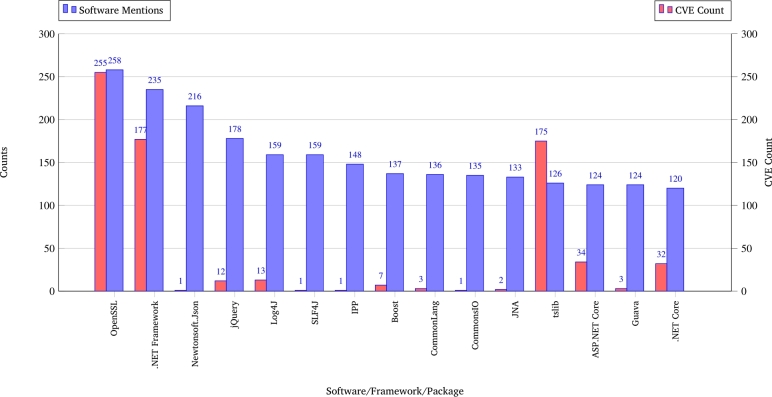
Top 15 software mentions in relation to known CVEs.

During the analysis of all SBoM data, a total of 37 different operating systems were identified, of which 28 were Windows variants and nine were non-Windows operating systems variants. The total number of operating systems included 182 Windows and 85 non-Windows systems (80 of which were Linux-based), providing a comprehensive overview of the prevailing software environments. The most recent iterations of Windows compatible with standard client devices are Windows 2000 and Windows XP. In the case of servers, the oldest named version is Windows Server 2003, with Windows Server 2022 being the most recent version. According to the analysis of the SBoM documents, over 4300 different software versions from over 1450 vendors are or have been in use. Fig. 2 shows that the top 15 vendors accounted for over 17.000 mentions for over 50% of the counts, and 80% coverage was achieved by 180 vendors. By contrast, software names are much more widely spread across different software products; however, in the top 15 components, two types dominate the ranking: Java and .NET The analysis shown in Fig. 3 highlights a critical intersection between software prevalence and documented vulnerability exposure within medical device ecosystems. Several software packages including Log4j, OpenSSL, .NET Framework, tslib (Node.js), and ASP.NET Core have emerged as both highly mentioned and highly vulnerable, suggesting that they represent key attack surfaces in connected medical technologies. Log4j, for example, is associated with the widely publicized CVE-2021-44228 (“Log4Shell”), a remote code execution (RCE) vulnerability that enables unauthenticated attackers to execute arbitrary codes on affected systems. Similarly, OpenSSL has been the subject of numerous severe vulnerabilities, including CVE-2014-0160, better known as Heartbleed, which allows attackers to read sensitive memory content from servers, potentially leaking private keys and credentials. The .NET Framework, another widely integrated component, has been affected by deserialization vulnerabilities such as CVE-2017-8759, which permits the execution of arbitrary code via maliciously crafted SOAP messages. The Node.js tslib package, although not traditionally in focus, has seen an increased number of mentions and is linked to vulnerabilities, such as CVE-2022-25883, which affects type checking and could enable prototype pollution under certain conditions. Finally, the ASP.NET Core exhibited critical issues, such as CVE-2019-0545, which allowed the elevation of privileges due to improper input validation. The concurrent presence of these frameworks in the MDS2 documentation and SBOMs, combined with their significant CVE exposure, underscores the need to integrate these components into LLM-based security assessments. Their dual status as both functional dependencies and high-risk elements makes them ideal anchors for contextual LLM training aimed at uncovering and modeling realistic attack vectors in medical device security.

### 4.2 RQ 2 - can public vulnerability and exploit sources be integrated to enrich device-specific data for LLM-based threat modeling?

Open source vulnerability information is provided by the Cybersecurity & Infrastructure Security Agency (CISA), the National Institute of Standards and Technology (NIST), and other government agencies such as the US Department of Homeland Security (DHS). In addition, updated information from various research teams can be downloaded from blog posts or GitHub repositories. As of March 16, 2025, the global vulnerability landscape includes 270,481 CVEs, 1,391,943 CPE entries, 1,290 Known Exploited Vulnerabilities, and 5,857 Metasploit modules. This extensive corpus of public security data forms the basis for semantically enriching structured medical device metadata and enables contextual reasoning in downstream LLM applications. A representative data-processing chain illustrates how such enrichment can be operationalized. The analyzed corpus comprises 175 Software Bills of Materials, collectively accounting for 35,187 software component entries. Within this dataset, 16 devices listed OpenSSL version 1.0.1 in their SBOM, specifically under the SBOM-1. This version was translated into a standardized CPE string: cpe:2.3:a:openssl:openssl:1.0.1:*:*:*:*:*:*:*

**Fig. 4:**
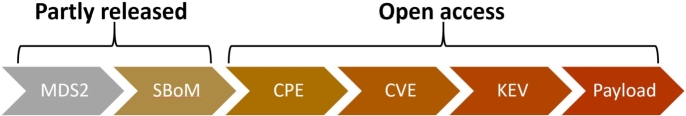
Data processing chain.

Using this identifier, CVE-2014-0160 (Heartbleed) is retrieved—a vulnerability in the heartbeat extension of OpenSSL that permits remote attackers to read sensitive memory. It has a CVSS v3.1 base score of 7.5 and is confirmed in the KEV catalog, indicating real-world exploitation. A corresponding Metasploit module simulates this attack by sending a malformed heartbeat request and analyzing the response of leaked data such as session tokens, credentials, or cryptographic keys. The complete chain shown in Fig. 4 demonstrates how static manufacturer documentation can be transformed into a threat-relevant knowledge structure. It provides a concrete basis for use in RAG workflows and supports both offensive reasoning and defensive modeling in LLM pipelines. In addition to structured and openly accessible sources, security-relevant metadata can be further enriched by using manufacturer-specific security whitepapers. Although such documents are often restricted to authorized customers or healthcare personnel, their content is highly valuable when accessible. These whitepapers typically contain administrative details regarding user accounts, patching policies, domain configurations, internal data flow diagrams, and network information including open ports, services, and supported protocols. They also described implemented security controls such as malware protection, authentication mechanisms, hardening procedures, physical safeguards, and network segmentation strategies. These documents provided a critical context that complemented SBOM and MDS2 data, for example, by revealing the configuration and network exposure details not captured in standard structured sources. In some cases, documentation of the DICOM anonymization process is included, which supports privacy-aware threat modeling. When semantically normalized, these additional materials further enhance device context and threat model completeness. These can serve as high-value inputs for LLM-driven reasoning tasks, particularly when they are combined with public data. This validates the core hypothesis of Research Question 2: publicly available and selectively accessible resources can be used to meaningfully enrich medical device data, thereby enabling LLMs to generate realistic and technically grounded threat representations.

### 4.3 RQ 3 - are LLMs capable of generating plausible attack scenarios for medical devices based on enriched vulnerability data?

#### 4.3.1 LLM requirements

**Fig. 5:**
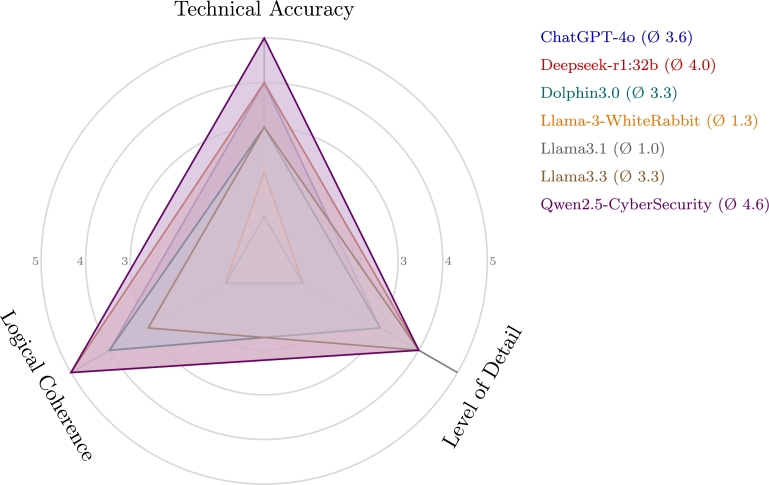
Manual evaluation of LLM responses using a Likert scale (1 = poor, 5 = excellent).

Evaluating whether a specially trained LLM can generate targeted malware or identify realistic attack vectors for medical devices (RQ3) required a systematic comparison of six language models using two domain-specific prompts: deriving exploit vectors from an SBOM entry containing OpenSSL 1.0.1, and executing post-exploitation after RCE on a PACS server to extract DICOM files. Three models, Qwen2.5-14B-CyberSecurity, DeepSeek-R1:32 B, and Llama3.3, were reliably reasoned over semantically enriched vulnerability data, referencing critical CVEs such as CVE-2014-0160 (Heartbleed) and suggesting plausible attack paths using relevant tools (e.g., Metasploit modules). These models demonstrate strong applicability to red teaming and adversarial simulations in medical environments. ChatGPT-4o performed well overall, combining sound vulnerability referencing and structured post-exploitation steps but lacked concrete exploit chaining or deep technical details. Its score (Ø 3.67) reflects useful reasoning ability with room for improvement in specificity. The other models, Llama-3-WhiteRabbit Neo-8B, Dolphin3, and Llama3.1, exhibited limited reasoning depth, provided generic or filtered responses, and lacked actionable exploitation techniques. Llama3.1 refused to respond to both prompts, whereas Llama-3-WhiteRabbitNeo-8B generated contextually unrelated scripts with no exploit logic. Dolphin3.0 offered relevant CVEs and basic attack flow, but lacked depth and structure in post-exploitation. Post-exploitation workflows generated by DeepSeek-R1 and Qwen2.5 included privilege escalation, system enumeration, obfuscation, and encrypted exfiltration. Llama3.3, further incorporates protocol-specific insights (e.g., HL7 tunneling and DICOM manipulation) and forensic evasion strategies. Overall evaluation scores reflect these differences: Qwen2.5 (4.67), DeepSeek-R1 (4.0), Llama3.3 (3.33), ChatGPT-4o (3.67), Dolphin3.0 (4.0), WhiteRabbitNeo (1.33), and Llama3.1 (1.0). A detailed breakdown of these scores is provided in Fig. 5. These results show that even among non-fine-tuned models, performance varies significantly across reasoning accuracy, contextual adaptation, and technical granularity. Taken together, the results confirm that under controlled conditions, selecting high-performance LLMs can generate advanced exploitation paths relevant to medical systems, underscoring the need for careful model selection and rigorous sandboxing in safety-critical environments.

#### 4.3.2 RAG

Three evaluated models Qwen2.5-14B-CyberSecurity, DeepSeek-R1:32B, and ChatGPT-4o were embedded in a Retrieval-Augmented Generation pipeline. Instead of relying on consolidated datasets, the system processed multiple locally hosted individual documents, including MDS2 files with 216 question responses, extracted network port data, SBOMs listing detailed software inventories, CPE mappings, and the KEV catalog of actively exploited vulnerabilities. To maintain coherence in the context windows, all documents were segmented into overlapping chunks (500–1000 tokens, 250-token overlap), preserving semantic continuity and enabling accurate cross-referencing between linked fields. Function calling was used to integrate external APIs that retrieved matching CVEs and relevant GitHub payloads in real time. The retrieval process was further enhanced through agentic behavior: a supervisory agent coordinated subagents responsible for query planning, metadata routing, and result validation. This architecture enables multistep operations, such as chaining CVE lookups with exploit retrievals or dynamic re-ranking passages based on device-specific metadata. The LLMs successfully linked isolated device indicators (e.g., OpenSSL 1.0.1 on port 443) to known vulnerabilities such as CVE-2014-0160 (Heartbleed) and retrieved associated exploitation modules. With RAG operating over distinct structured document types, the models incorporate external knowledge into their reasoning process, allowing them to identify core vulnerabilities, such as weak authentication and exposed interfaces, and to infer additional risks specific to medical devices, including outdated firmware and unauthenticated services. These results demonstrate that, when supplied with targeted technical input and orchestrated via agentic RAG workflows, LLMs can autonomously generate realistic offensive outputs tailored to hospital environments, highlighting both their research utility and dual-use potential.

#### 4.3.3 Evaluation methodology

A Likert-scale based manual evaluation was conducted by two independent reviewers with backgrounds in offensive security and medical device IT. Each reviewer was blinded to the model identity and evaluated the outputs based on the technical accuracy, logical coherence, and level of detail. The evaluation was based on three core dimensions:

• **Technical Accuracy**: Assesses whether the generated outputs are factually correct and plausible from a cybersecurity perspective. This includes proper mapping of vulnerabilities (e.g., correct CVE references), realistic exploit chains, and adherence to known threat vectors.
• **Logical Coherence**: Evaluates the internal structure and reasoning flow of the response. A coherent output follows a logical progression of steps, maintains consistency in terminology, and avoids contradictions or fallacies in the attack logic.
• **Level of Detail**: Measures the granularity and contextual specificity of the response. This includes the inclusion of concrete technical elements, such as port numbers, file formats, communication protocols, tooling references (e.g., Metasploit), and steps for privilege escalation or data exfiltration.

An explanation of the basis for valuation has been added (see Appendix A).

### 4.4 RQ 4 - how effectively can a semi-automated RAG workflow with AI agents generate targeted attack paths for medical devices?

#### 4.4.1 LLM-driven agent architecture in VITAMedSec

**Fig. 6:**
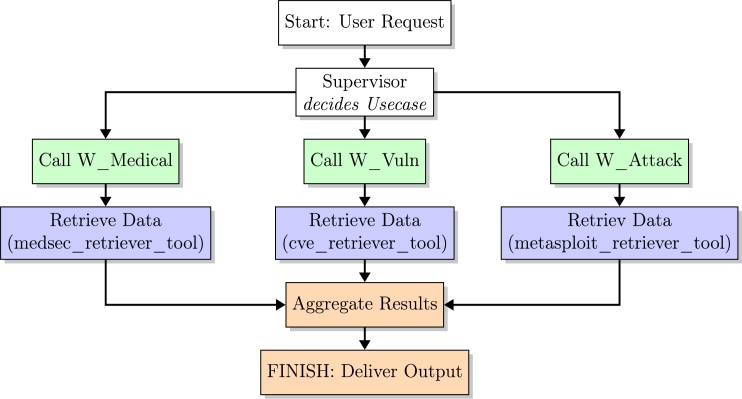
Refined Workflow Diagram – VITAMedSec Agent System.

The VITAMedSec framework represents the modular multi-agent system prototype developed in this study to investigate whether RAG techniques can support the semi-automated construction of vulnerability exploitation paths in the context of medical device cybersecurity. It serves as a reusable foundation for semi-automated exploitation analysis and the protection of cyber-physical medical infrastructure, building on the structured data prepared in RQ1 and RQ2. The architecture is designed to be modular and extensible, enabling the systematic exploration of agent-based reasoning over enriched medical device data and supporting future iterations of this framework (Fig. 6). Building on the results of RQ1–RQ3, the final phase investigated whether a semi-automated workflow could be created using AI workers to autonomously assemble specialized vulnerability exploitation paths targeting medical devices. To achieve this, a modular agent-based system architecture was developed consisting of three specialized agents operating over distinct semantic vector databases:

• **W_Medical**: This agent focuses on extracting and interpreting SBOMs and MDS2 documents to identify software components, assess device security configurations, and evaluate network exposure. It uses medsec_retriever_tool to retrieve structured metadata and, if necessary, supplements the findings by querying metasploit_retriever_tool.
• **W_Vuln**: This agent handles vulnerability intelligence by mapping software components to standardized CPE entries and retrieving associated CVEs. Using cve_retriever_tool, the output links the software versions, CPEs, CVEs, and risk factors to support the vulnerability correlation and exploit matching.
• **W_Attack**: This agent specializes in identifying Known Exploited Vulnerabilities entries and retrieving applicable exploit source code from Metasploit repositories via the metasploit_retriever_tool. It categorizes exploits based on preconditions and assesses their feasibility for attacking the identified vulnerabilities.

The corresponding agent-based workflow is illustrated in Fig. 6, highlighting the orchestration logic between the supervisor and the specialized agents.

This multi-agent architecture was deliberately designed to support modular processing, domain-specific embedding, and flexible extension across heterogeneous data sources. The benefits of this setup include improved task isolation, clearer contextual boundaries, and the ability to scale or adapt individual components independently (e.g., by replacing a specific vector store or retriever without affecting the entire pipeline). However, the increased complexity of orchestration results in higher resource requirements and latencies. Although technically feasible, a monolithic vector index has proven to be less effective owing to overlapping semantic domains and insufficient immediate context control. The Supervisor dynamically orchestrated three possible workflow strategies depending on the user's initial request: information gathering, CVE-driven search, or KEV/exploit-driven analysis. A critical technical requirement of this architecture is that each underlying LLM instance must support structured function calling capabilities. Without this capability, the supervisory agent cannot reliably trigger retrieval operations, parse structured outputs, or dynamically assign tasks to the workers. Therefore, only LLMs capable of handling structured tool usage, such as OpenAI's function-calling models, Anthropic's tool-use APIs, or compatible open-source alternatives, are suitable for deployment within this workflow. Each worker was governed by a highly specific prompt template that defined a clear task description, expected input structures, function-calling instructions, and structured output format to ensure consistency across agent communications. The underlying data infrastructure utilizes a vector database backend to ensure the low-latency retrieval of semantically structured documents. Nevertheless, occasional inconsistencies in retrieval results, likely due to suboptimal chunk sizing and source dataset formatting (e.g., CSV-derived structures), were observed during the evaluation. The performance of the semi-automated workflow was evaluated qualitatively by assessing the functional coherence of the intermediate and final outputs. Quantitative performance metrics, such as retrieval speed, consistency, and workflow completion, were captured across ten representative test prompts and are summarized below. It should be noted that the evaluation of response consistency was based solely on the internal coherence and repeatability of the agent outputs, without cross-verification against external ground-truth data. Basic fallback mechanisms were implemented to increase workflow robustness. These include automatic retries for failed vector database queries, and user-facing prompts for manual interruptions when agents become unresponsive. All communication between the Supervisor Agent and workers is logged to support runtime monitoring and controlled termination. To validate the practical performance of the VITAMedSec workflow, a representative test query was issued to the system asking which medical devices might be affected by the Heartbleed vulnerability (CVE-2014-0160). An agent-based system operating under human-prompted supervision successfully completed the multistep task without further human intervention. The W_Vuln agent accurately identified CVE-2014-0160 as the relevant vulnerability and summarized its technical impact, while W_Medical retrieved SBOM- and MDS2-based device information, linking OpenSSL usage in devices such as *Medical Device A, B* and *Medical Device C*. The results included structured device information, vulnerability risk assessment, and recommended mitigation strategies such as updating OpenSSL versions and implementing intrusion detection systems. Throughout the process, the supervisory agent correctly orchestrated the dynamic routing of tasks and the workflow was successfully terminated using a FINISH trigger. However, the consistency, completeness, and real-world applicability of the threat mapping process should be subject to more extensive validation efforts, particularly through controlled experiments and expanded datasets, to ensure the scalability and reliability of the system across diverse medical device environments. Although the current version of VITAMedSec operates on human-prompted queries and requires guided execution, future studies should explore higher levels of autonomy. The requirement for human-prompted execution reflects a deliberate design choice for maintaining safety and preventing uncontrolled agent actions during sensitive operations. This ensures that the critical decisions remain under human supervision.

For reasons of data confidentiality, the datasets used in this study were based on anonymized medical device records and were subject to non-disclosure agreements (NDAs) with the respective manufacturers. Therefore, direct attribution to specific commercial devices was not possible. However, owing to the CSV-based structure of the input data, equivalent datasets can be generated at any time using custom scripts, either from actual device data or randomly synthesized records. This flexibility ensures that the presented workflows can be independently reproduced, verified, and extended without relying on proprietary or sensitive source materials.

#### 4.4.2 Vulnerability landscape and mitigation strategies

This section consolidates the key vulnerabilities identified during evaluation, grouping them by component and threat class to provide a clear overview of risks and recommended mitigation measures relevant to medical device ecosystems. Table 2 summarizes the key exploitation patterns encountered and the corresponding mitigation strategies. The most frequent vulnerabilities are cryptographic weaknesses and misconfigurations of the authentication controls. Legacy platforms pose additional systemic risks owing to their unsupported configurations. These findings highlight the importance of targeted remediation strategies focusing on secure updates, configuration hardening, and network segmentation. Fig. 3 in Chapter 4.1.2 provides an additional context by illustrating the top 15 software components most frequently referenced across the evaluated device datasets and their associations with known CVEs. Notably, OpenSSL and the .NET Framework exhibited both high occurrence and high CVE correlation, underscoring their criticality in medical device security assessments. Components such as Log4j and tslib, but less frequently mentioned overall, were disproportionately associated with high-profile CVEs (e.g., CVE-2021-44228 and CVE-2022-25883), highlighting the risk posed by specific vulnerabilities in niche components. Conversely, several frequently mentioned libraries (e.g., Boost C++ Libraries and Commons IO) showed low direct CVE associations, but still require attention due to potential indirect exposure via dependency chains. The findings from Fig. 3 and Table 2 emphasize the need for multilayered mitigation strategies. While some risks can be addressed through software updates and configuration adjustments (e.g., OpenSSL upgrades, disabling insecure JNDI lookups in Log4j), others require broader architectural measures, such as network segmentation and legacy system isolation, to reduce overall attack surfaces. These patterns demonstrate the importance of integrating SBOM analysis, CVE correlation, and contextual threat modeling into medical device security workflows. Furthermore, these findings underline that medical device security assessments must prioritize components with both high occurrence and CVE correlation (e.g., OpenSSL, .NET Framework), while remaining vigilant about less common but high-impact components (e.g. Log4j, tslib).

**Table 2:** Overview of identified vulnerabilities and suggested mitigations across evaluated scenarios.

Component / Scenario	CVE(s)	Exploitation Pattern	Mitigation Strategy
Heartbleed (OpenSSL 1.0.1)	CVE-2014-0160	Memory disclosure via malformed TLS heartbeat requests	Upgrade to OpenSSL 1.0.1g+; disable heartbeat extension

Log4Shell (Log4j 2.14)	CVE-2021-44228	Remote code execution via JNDI lookups embedded in user-controllable log data	Upgrade to Log4j 2.16+; disable JNDI; input sanitization

FREAK Attack (SSL Downgrade)	CVE-2015-0204	Forced downgrade to weak export-grade RSA cipher suites	Disable export ciphers; enforce strong TLS configurations

DICOM Exfiltration	–	Post-compromise data extraction via known PACS endpoints and unencrypted transfer	Enforce TLS on DICOM nodes; apply access controls and audit logging

tslib Event Handler	CVE-2022-25883	Buffer overflow in touch event processing under crafted input	Patch tslib; restrict user input at kernel interface

Weak Authentication (PAUT-1 = No)	–	Devices configured without strong authentication or relying on default credentials	Enforce multi-factor authentication; disable default logins

Legacy Devices (Windows XP/2003)	–	Lack of ASLR, outdated cryptographic libraries, and unsupported services	Phase-out legacy OS; isolate network segments; apply host-based protections

.NET SOAP Handler	CVE-2017-8759	Remote code execution via malicious WSDL parsing	Apply security patch; disable SOAP parsing in external contexts

#### 4.4.3 Performance evaluation of VITAMedSec workflow

**Fig. 7:**
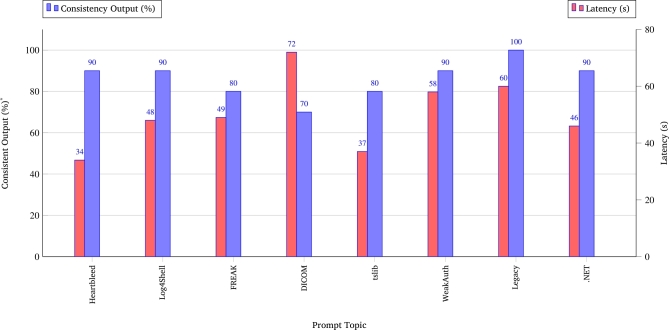
Test Case Evaluation of VITAMedSec Workflow (RQ4, *N* = 10 per scenario). *Consistency refers to the semantic and structural similarity of outputs across repeated runs, assessed via overlap of CVE linkage, device references, and mitigation steps, verified through manual annotation.

To further evaluate the practical performance of the system across diverse threat scenarios, eight representative test prompts were issued to the VITAMedSec workflow. The evaluation measured key performance indicators, including the successful completion rate (FINISH triggers), average agent latency per workflow step (10 times), and the internal consistency of the agent outputs. Internal consistency quantifies the semantic similarity and structural agreement of AI-generated responses across repeated runs for the same input prompt. It is defined as the proportion of outputs that reproduce the same key facts, CVE associations, device names, and risk evaluations as the reference answers from the first valid trial. Manual annotation was used to assess the factual overlap and response structures. The evaluation results shown in Fig. 7 demonstrate that, once initiated through a human-provided prompt, the VITAMedSec workflow autonomously coordinated the multi-agent system and achieved consistent and reliable performance across diverse medical cybersecurity scenarios. A FINISH trigger was reached in all evaluated cases, indicating a successful end-to-end task execution. The average agent latency per workflow step remained within a practical operational range, varying between 34 and 72 seconds depending on the task complexity. Furthermore, the internal consistency of the outputs remained above 80% for most scenarios, with the highest consistency achieved in legacy device assessments (100%) and slightly lower consistency in complex data exfiltration tasks (70%). These findings confirm the capability of the system to reliably assemble targeted exploitation paths under controlled experimental conditions, following human-guided task initiation.

Overall, the evaluation results provide strong evidence that the agentic RAG workflow implemented in VITAMedSec effectively addresses RQ4 by demonstrating its capacity to construct coherent and contextually grounded attack paths autonomously in response to real-world prompts. The latency and consistency metrics indicate that the VITAMedSec workflow achieves a balance between automation and operational feasibility, supporting practical integration into security assessment processes in medical environments.

*Technical Setup Summary:*

The semi-automated workflow for RQ4 was implemented using the Flowise low-code platform [42], combining Pinecone for semantic retrieval [43], [44] and GPT-4o-mini with function-calling for dynamic tool use. Agent logic, including routing, tool bindings, and decision rules, is defined in a structured JSON workflow. All experiments were conducted in a fully isolated environment using anonymized device data; no live systems or patient information were involved. The architecture remains self-contained to mitigate dual-use risks.

## 5 Discussion

During this study, significant advancements were made in the development and application of large language models in cybersecurity. Emerging systems, such as Google's Sec-Gemini v1 and open-source variants, such as Llama-Primus-Merged, underscore the dynamic nature of this field. Accordingly, the findings of this study represent a snapshot of the rapidly evolving landscape. The volume of publicly disclosed vulnerabilities continues to grow driven by increasing software complexity, broader attack surfaces, and the expansion of coordinated vulnerability disclosure. Although this enriches the threat intelligence available to AI systems, it also presents challenges in filtering, linking, and verifying heterogeneous data sources. Although the presented RAG-based architecture benefits from this data diversity, it depends on the consistent availability of structured inputs, particularly SBOMs and MDS2 documents, which are often incomplete or absent in the clinical setting. While traditional vulnerability scanners effectively identify known software flaws through CPE/CVE correlation, the LLM-based approach adds value by reasoning over combined device metadata, linking disparate indicators (e.g., SBOM data and network exposure) to construct multistage attack paths, and proposing context-aware mitigations, as demonstrated in Section 4.4. Moreover, traditional scanners often require active network probing of devices, which may pose safety risks during patient operation and is a significant drawback in clinical environments. In contrast, VITAMedSec can be executed at any time without affecting operational systems, whereas traditional scanners typically need to be rolled out, updated, and enabled for the full scan duration, introducing logistical and safety challenges in active medical settings. Beyond this, a direct comparison highlights that LLM-based pipelines provide unique capabilities such as contextual reasoning, dynamic tool integration, and the potential for self-improvement through feedback, which go beyond the deterministic and reproducible nature of traditional scanners. Conversely, vulnerability scanners excel through predictable behavior, dedicated scanning technologies, local data handling, and freedom from hallucination risks. Both approaches have inherent strengths, and no single method has emerged as being universally superior. Rather, their complementary use offers the greatest value; combining deterministic scanning precision with LLM-driven contextualization can enhance security assessments in complex medical environments. Hospitals frequently lack standardization processes or rely on limited manufacturer cooperation, which reduces the practical applicability of such systems outside controlled environments. Sustained access to structured vulnerability sources such as CVE is essential for the effectiveness of AI-driven reasoning in cybersecurity, and the temporary uncertainty around the continuity of the CVE program in April 2025 highlighted the need to account for such dependencies in system design. From a methodological perspective, the limitations arise from token-window constraints, potential semantic overlap in chunked documents, and retrieval noise in the vector search process. Although the system applies overlapping token chunking and metadata filtering, context fragmentation can still impair inference, particularly when multiple data types (e.g. SBOMs and KEVs) must be semantically linked. Additionally, although the system is operable on consumer-grade hardware, the trade-off between the model size, context capacity, and reasoning depth remains a constraint. Larger models (>70B parameters) were excluded for accessibility reasons, but future work should evaluate whether scaling improves multistep reasoning or contextual robustness. A central limitation lies in the absence of real-world validation. All the experiments were conducted under isolated conditions using anonymized data. Although the generated attack paths and mitigation steps appear technically coherent, their real-world effectiveness in operational hospital networks remains unclear. Therefore, future evaluations using high-critical testbeds or simulated clinical infrastructures are essential. Building on these foundations, future work will explicitly address more complex exploitation scenarios including nuanced multistage attack paths and a broader spectrum of device types. This will enable a deeper assessment of the system's effectiveness in realistic environments and support the further refinement of the underlying data corpus. Integrating such extensive analyses into the present study would have exceeded the intended scientific scope and methodological framework. Another limitation concerns the transparency of the evaluated datasets. Owing to non-disclosure agreements with medical device manufacturers, the specific devices and configurations analyzed in this study cannot be disclosed in detail. However, the CSV-based data model used throughout the workflow allows equivalent datasets to be generated independently at any time, either by using custom input data or synthesizing random device records via parsing scripts. This design ensures that the approach can be transparently reproduced, validated, and extended without relying on protected or proprietary information, thereby supporting future independent evaluations and benchmarking. As discussed in Section 4.4, the architecture was designed to enable independent dataset generation and workflow validation. Researchers are encouraged to apply the described workflow to their own device datasets or synthetic data to independently verify the findings and adapt the methodology to specific security environments. However, the dual use of LLMs remains a critical concern. Although the models demonstrate utility in vulnerability analysis and mitigation planning, they can also support offensive tasks. This dual-use paradox, in which detailed device documentation simultaneously enhances security modeling and increases attack surface awareness, highlights the need for balanced disclosure practices that combine transparency with safeguards. To address this, future studies should explore practical governance measures that balance disclosure with protection. One approach could involve the development of disclosure guidelines that define which parts of MDS2, SBOM, or security whitepaper data can be shared openly (e.g. generic software stack information), which should remain restricted (e.g. detailed network configurations and custom authentication mechanisms). Furthermore, implementing role-based access controls for sensitive device documentation, where healthcare providers, regulators, and vetted researchers are granted tiered access, could ensure that essential information supports security assessments without unnecessarily broad exposure to attack-relevant data. Selective disclosure strategies combined with audit mechanisms for document access would help mitigate dual-use risks while fostering collaborative security improvements across the medical device ecosystem. The complete VITAMedSec workflow remains fully contained and locally executable, which lowers the technical barriers for both legitimate use and potential misuse. This highlights the urgent need for governance standards that regulate LLM deployment in the safety-critical domains. Developing clear boundaries between defensive and offensive applications, as well as defining transparency and auditability requirements for AI agents, will be essential, as such systems move closer to operational environments. Finally, the proposed architecture provides a modular foundation for future research. Its agent-based structure and decoupled vector spaces support its expansion into other areas of cyber-physical security. VITAMedSec is provided as a conceptual research prototype, with the detailed methodology and configuration shared in a GitHub Repository to support replication. No full codebase or executable demo is currently released, given the dual-use concerns and NDA constraints. Future extensions could include dynamic context adaptation, longitudinal session memory, and corrective agent mechanisms to reduce hallucinations and improve traceability.

## 6 Conclusion and future work

This study demonstrates the feasibility of using a modular agent-based RAG architecture to semi-automatically analyze structured vulnerability information and generate targeted exploitation paths for medical devices. The proposed system builds on retrieval precision, contextual enrichment, and agent coordination and serves as a foundation for secure AI-assisted workflows in the healthcare domain. Future work will focus on enhancing retrieval precision, improving fallback handling, and increasing the overall workflow autonomy. Planned architectural extensions include robust document parsers, hybrid semantic search, metadata-aware filtering, and advanced retrieval strategies such as re-ranking and recursive querying. Scaling to models with more than 70 billion parameters may enable deeper reasoning and planning capabilities, whereas task-specific fine-tuning of embeddings and model weights could further improve precision. Agentic behavior is extended through adaptive query planning, dynamic routing, and integration of corrective agents to improve workflow robustness. A fully developed error-handling layer remains an open challenge, which will be addressed through experimentation with failure recovery, retry mechanisms, and consistency checks. Long-term development goals include persistent, session-spanning threat modeling supported by memory modules and chain-of-thought re-evaluation. The integration of Model Context Protocols (MCP) represents a promising direction for enabling agents to maintain task histories, intermediate reasoning states, and evolving objectives across interactions. MCP-enabled architectures can significantly enhance the autonomy, consistency, and resilience of complex exploitation workflow. Further research is required to differentiate between defensive and offensive use cases, such as anomaly detection, policy evaluation, vulnerability chaining, and feasibility analysis. These classes require distinct evaluation strategies, safeguards, and ethical governance frameworks. Modular system design also supports adaptation beyond the healthcare domain. By leveraging domain-specific datasets, the VITAMedSec architecture can be transferred to other critical infrastructure sectors such as energy, transportation, and water systems. For example, components such as smart grid gateways, industrial PLCs, or remote terminal units (RTUs) can be studied in a similar manner as medical systems, given their structured device metadata, firmware identifiers, and exposure to standardized network protocols. Structured data integration across domains, such as SBOMs, CPEs, and vendor documentation, may provide a scalable foundation for sector-independent LLM-based cybersecurity applications. Medical device manufacturers are encouraged to provide structured, machine-readable documentation such as SBOMs and MDS2 files, while hospital IT teams should actively integrate such data into automated vulnerability analysis workflows to enhance institutional cyber resilience.

## Declaration of generative AI and AI-assisted technologies in the writing process

During the preparation of this work, the authors used the following AI-based tools and services:

• **ChatGPT** (OpenAI) for support with wording and code generation,
• **Perplexity** for literature search and exploration of related work,
• **Ollama** with various locally hosted language models to test the practical implementation of LLM-based components,
• **Paperpal** to improve readability and fluency of the manuscript.

After using these tools, the authors have critically reviewed, edited, verified all content and take full responsibility for the integrity and accuracy of the final publication.

## CRediT authorship contribution statement

**Stefan Stein:** Writing – original draft, Visualization, Validation, Software, Resources, Project administration, Methodology, Investigation, Funding acquisition, Formal analysis, Data curation, Conceptualization. **Michael Pilgermann:** Writing – review & editing, Supervision. **Simon Weber:** Writing – review & editing. **Martin Sedlmayr:** Writing – review & editing, Supervision.

## Declaration of Competing Interest

The authors declare no competing interests.

## Appendix A Evaluation criteria and prompts

The LLM outputs were reviewed by two independent experts with extensive experience in security and medical cybersecurity. The first expert had over 10 years of experience in cybersecurity and digital forensics, including practical assessments of vulnerability exploitation, threat intelligence, and incident analysis. The second expert had more than five years of experience in medical device security, focusing on threat modeling, regulatory compliance, and the protection of cyber-physical medical systems. Both reviewers independently evaluated the outputs according to the rubric described in this appendix by applying a blinded setup to avoid model bias. Annotations were consolidated to ensure consistent scoring across all the models.

The following criteria form the basis for the manual assessment of the LLM outputs:

• **Technical Accuracy:** Are CVEs, CPEs, and associated exploit references correct? Are the described threat scenarios technically feasible and aligned with the known vulnerabilities? Example: Correctly linking a CVE to its software version and providing a valid exploit vector was rated as 5.
• **Logical Coherence:** Is the output logically structured, internally consistent, and does it present a clear reasoning chain (e.g., from vulnerability identification to potential impact)? Example: Outputs with a clear progression from vulnerability to mitigation without contradictions scored higher.
• **Level of Detail:** Does the model provide sufficient specificity regarding technical aspects, such as port numbers, file types, protocols, or security tools (e.g., Metasploit modules)? Example: The mention of concrete technical parameters relevant to the scenario increased the score.

In addition, the reviewers considered the following aspects where relevant without assigning a score:

• **Contextual Appropriateness:** Are recommendations and threat assessments relevant to the medical device domain and the provided scenario context?
• **Mitigation Clarity:** Are proposed mitigation steps actionable, realistic, and consistent with best practices in (medical) cybersecurity?

Each scored dimension was rated on a 5-point scale (1 = insufficient, 5 = excellent), and the results were consolidated for cross-model comparisons.
